# Preconception expanded carrier screening: a focus group study with relatives of mucopolysaccharidosis type III patients and the general population

**DOI:** 10.1007/s12687-021-00519-2

**Published:** 2021-03-22

**Authors:** Thirsa Conijn, Ivy van Dijke, Lotte Haverman, Phillis Lakeman, Frits A Wijburg, Lidewij Henneman

**Affiliations:** 1 Amsterdam UMC, Pediatric Metabolic Diseases, Emma Children’s Hospital and Amsterdam Lysosome Center “Sphinx”, University of Amsterdam, Amsterdam, the Netherlands; 2 Amsterdam UMC, Psychosocial Department, Emma Children’s Hospital, University of Amsterdam, Amsterdam, the Netherlands; 3 Amsterdam UMC, Department of Clinical Genetics, Amsterdam Reproduction and Development Research Institute, Vrije Universiteit Amsterdam, PO Box 7057, 1007 MB Amsterdam, the Netherlands; 4 Amsterdam UMC, Center for Reproductive Medicine, University of Amsterdam, Amsterdam, the Netherlands; 5 Amsterdam UMC, Department of Clinical Genetics, Amsterdam Reproduction and Development Research Institute, University of Amsterdam, Amsterdam, the Netherlands

**Keywords:** Expanded carrier screening, Genetic screening, Perspectives, Mucopolysaccharidosis type III, Focus groups, Qualitative study

## Abstract

Preconception expanded carrier screening (ECS) enables prospective parents to assess their risk of having a child with an autosomal recessive disorder. Knowledge on motivations, feelings, and considerations people have towards the offer and use of ECS is limited. To enrich the public and professional discussion on ECS implementation, this study explored the perspectives towards various aspects of ECS in seven focus groups compromising first- and second-degree relatives of MPS III patients (N=9, N=4, N=5, N=5) and members of the general Dutch population (N=6, N=7, N=5). The focus groups were audio recorded and the transcripts were qualitatively analyzed to identify themes. Both relatives of MPS III patients and participants from the general population supported offering ECS, in particular for severe, childhood-onset disorders. Important barriers identified for ECS were a lack of genetic knowledge and a perceived lack of personal relevance and awareness, as well as out-of-pocket costs of testing. The majority of participants would prefer full disclosure of individual test results instead of couple-based test results. Moreover, offering people a choice for the way of reporting was proposed. All participants agreed that more efforts, for example by governmental campaigns, should be made to increase awareness on the availability, potentials, and limitations of ECS. Educating prospective parents about ECS is essential for increasing awareness and informed decision making. This study provides valuable insights that can be used by governments and public health authorities when considering implementation of preconception ECS.

## Introduction

Preconception carrier screening offers prospective parents the possibility to obtain information about their risk of having a child with an autosomal recessive (AR) or X-linked disorder before pregnancy (Edwards et al. 2015). Identified high-risk couples can consider a range of reproductive options such as preimplantation genetic testing (PGT), prenatal diagnosis, the use of donor gametes, and accepting the risk or refrain from having children (Henneman et al. 2016). Carrier testing for severe genetic disorders is conventionally aimed at couples with an a priori increased risk of having affected offspring based on family history, geographic, and/or ethnic background or consanguinity (Bennett et al. 2002; Edwards et al. 2015; Henneman et al. 2016). Next-generation sequencing (NGS) allows for testing for multiple AR disorders simultaneously without significantly higher costs, thereby increasing the feasibility of offering universal expanded carrier screening (ECS) panels to the general population (Wilfond et al. 2018). Several commercial companies in the USA, Australia, and Europe now offer ECS directly to consumers, often without appropriate genetic pre- and post-counselling (Chokoshvili et al. 2018a). Recently, non-profit health organizations in Australia (Delatycki et al. 2020), Belgium (Borry et al. 2017), and the Netherlands (Nijmeijer et al. 2019; Plantinga et al. 2016) started to initiate ECS for individuals without an a priori increased risk.

In the Netherlands, there are no private providers offering carrier testing and within general healthcare a standard offer of ECS to prospective parents is lacking (Delatycki et al. 2020). Two academic medical centers developed a non-commercial carrier screening test for 50–70 severe autosomal recessive disorders. These tests are available for all prospective parents in the Netherlands, paid on their own costs, but are in general not actively offered to them by healthcare professionals. The Amsterdam University Medical Centres (Amsterdam UMC) panel mainly comprises childhood-onset, severe inborn errors of metabolism (IEMs) for which no or only limited effective disease-modifying treatment is currently available, such as mucopolysaccharidosis type III (MPS III or Sanfilippo syndrome), Tay-Sachs disease, and Batten disease (Amsterdam UMC 2020).

In comparison to targeted screening, preconception ECS maximizes opportunities for autonomous reproductive choices by providing more genetic information and thus more equity of access to carrier testing (van der Hout et al. 2016). However, the implementation of ECS raises several practical, social, and ethical concerns, such as downstream medical service costs, psychosocial harms, and concerns about potential societal pressure to use ECS (Holtkamp et al. 2017; Kihlbom 2016; Kraft et al. 2019; van der Hout et al. 2016). Moreover, according to stakeholders, a lack of demand for genetic carrier screening in the general public is an important barrier for implementation (Holtkamp et al. 2017).

Several studies assessed the attitudes towards ECS among individuals and couples from the general population, suggesting that there is interest among the target population (Chokoshvili et al. 2017; Nijmeijer et al. 2019; Ong et al. 2018; Plantinga et al. 2016; Spencer et al. 2018; Van Steijvoort et al. 2020). Recently, the study of Nijmeijer et al. (2020) showed that relatives of patients with MPS III had more positive attitudes towards ECS than members of the general population, which is in line with the results of other studies assessing attitudes of patients and relatives towards carrier screening for, e.g., fragile X syndrome (Archibald et al. 2009; Skinner et al. 2003), spinal muscular atrophy (SMA) (Boardman et al. 2017), cystic fibrosis (CF) (Janssens et al. 2016; Maxwell et al. 2011), and hemophilia (Boardman et al. 2019). Involving patients and families in discussions about the implementation of ECS can provide important information, as they may perceive different issues on ECS than the general population (Boardman et al. 2019). These may include concerns about a decline in future funding of research for potential treatment options and the possible negative shift of public attitudes towards people with a disability (Kraft et al. 2019; Parens and Asch 2003).

Another aspect associated with the implementation of ECS is the large heterogeneity of ECS panels. The number of disorders included in current panels varies from 40 to almost 1500 disorders (Chokoshvili et al. 2018b; Rowe and Wright 2020). Although European (Henneman et al. 2016), American (Edwards et al. 2015), and Australian (Kirk et al. 2020) guidelines and pilot studies have proposed consensus-based criteria for the composition of panels, major differences were shown in an overview of 16 available (commercial) ECS tests with overlap in only three disorders for all providers (Chokoshvili et al. 2018b). While earlier studies gauged the opinions of (mainly) clinical geneticists or other healthcare workers on inclusion criteria for ECS panels (Chokoshvili et al. 2016; Kirk et al. 2020; Lazarin et al. 2014), little is known about the opinion of potential users of ECS or by (relatives of) patients affected with genetic disorders towards their desired composition of these panels.

Finally, there is no uniformity in the way how to disclose ECS test results to prospective parents. Results can be disclosed to partners individually (full disclosure of individual test results) or as a couple-based result (individual carrier states are only reported if a carrier couple is identified, i.e., both partners carry a pathogenic variant in the same gene) (Henneman et al. 2016; Plantinga et al. 2019). Surveys among the general population suggest that most individuals prefer full disclosure of individual test results (Nijmeijer et al. 2019), although most couples do not seem to object towards receiving couple-based results only (Plantinga et al. 2019). However, the considerations on which such preferences are based are unknown.

This qualitative focus group study aims to obtain insight into the perspectives of relatives of MPS III patients and potential users from the general population on ECS, including attitudes towards the offer of ECS, the composition of ECS panels, and the disclosure of test results (individual or couple-based), to enrich the discussion on the implementation of ECS. Relatives of MPS III patients were selected as this disorder meets all the international consensus-based criteria for the composition of ECS panels and is included in most ECS panels (Edwards et al. 2015; Henneman et al. 2016; Kirk et al. 2020).

## Methods

### Study design

This study used a focus group design as this enables a setting in which people’s feelings, perceptions, and reactions can be carefully assessed (Litosseliti 2003). Seven semi-structured focus groups were conducted between May 2019 and September 2019. The study was approved by the Medical Ethics Committee of the Amsterdam UMC, the Netherlands.

### Participants

Parents of all MPS III patients currently under treatment in the Dutch national expertise center received an invitation by email from a member of the research team (response rate 32%) and were subsequently asked to forward this email (introducing the topic) to other relatives, as we did not have their contact details. Therefore, we do not know this response rate. Relatives were compensated for their participation with a gift card of 25 euro and reimbursement of travel costs. First- and second-degree relatives participated in separate focus groups, resulting in three focus groups with first-degree relatives (group (Gr)#1, N=9/Gr#2, N=4/Gr#3, N=5), and one focus group with second-degree relatives (Gr#4, N=5).

Three focus groups (Gr#5, N=6/Gr#6, N=7/Gr#7, N=5) comprised individuals from the general population who were planning to have (more) children. Participants were recruited by CGselecties, a Dutch research marketing agency that provides a panel of more than 25,000 individuals who are willing to participate in (qualitative) research on a regular basis in return for an incentive of 45 euro. The sample was selected from their panel based on the demographics gender, regional area, educational level, and reproductive age to represent the Dutch population (response rate ~9%). Participants were not informed by the agency about the topic which would be discussed during the focus group.

### Focus group guide and procedure

All focus group meetings took place in a meeting room at the Amsterdam UMC and had a duration of approximately 2 h. Six out of seven focus groups were moderated by the same researcher (TC) and one focus group was moderated by another member of the research team (LHe). In each focus group, the moderator was assisted by another member of the research team (LHa, FAW, HvO, IvD).

Before the start of the focus group, the participants from the general population were shown an educational video (https://youtu.be/V9FKDNF_-tI) in which information on AR inheritance, the ECS test for 50 genetic disorders in the Amsterdam UMC as an example of an ECS test, reproductive options in case of an increased risk (1:4) of affected offspring, and a description of the nature and course of MPS III as an example of a disorder included in ECS panels were provided. The educational video was not shown to relatives as we know that visuals of other MPS III patients may elicit strong and undesired emotional responses in the setting of a focus group meeting, but they received a short presentation with similar information on AR inheritance, the ECS test for 50 genetic disorders, and reproductive options.

The focus group guide was developed by members of the multidisciplinary research team, including experts in psychology, clinical genetics, health sciences, and metabolic diseases. The guide consisted of four main topics: reasons (self or others) to accept or decline ECS using the Amsterdam UMC test as an example, awareness on the possibility of ECS, the composition of ECS panels, and preferences in disclosure of test results. Reasons (not) to opt for ECS were discussed and prioritized using post-its. Subsequently, the moderator presented a list of categories to enhance the discussion on the composition of ECS panels, consisting of severe life-threatening disorders that lack treatment, intellectual disability, physical disability, late-onset disorders, and the category “(prospective) parents are free to choose”. Although a focus group guide was composed, participants were also invited to discuss other topics related to ECS which they considered relevant.

At the end of each focus group session, participants completed a brief questionnaire assessing sociodemographic characteristics (age, gender, educational level, considering a (future) pregnancy, marital status, and religious beliefs). Two additional questions assessed familiarity with the Amsterdam UMC carrier test and previous experience with genetic carrier testing in general.

### Data analysis

Data collection stopped until data saturation was reached. All sessions were audio recorded and transcribed verbatim without any individually identifiable details to guarantee full anonymity of the participants. The transcripts were analyzed with a thematic coding method (Clarke et al. 2015). Two researchers (TC and IvD) independently analyzed the first five transcripts using qualitative software MAXQDA 2020 (VERBI Software, 2019). Relevant parts of the transcripts were marked and codes were generated to organize data into meaningful groups (data was assigned to only one code). Subsequently, themes were identified and codes were assigned to the identified themes. Differences in codes and themes were discussed until consensus was reached. TC subsequently analyzed the remaining two focus groups. The final thematic framework matrices were discussed with another member of the research group (LHe) until consensus was reached. Finally, the thematic frameworks of the focus groups with relatives of MPS III patients and individuals from the general population were contrasted and compared. Central themes were discussed and illustrated with quotations. For each theme, the most eloquent quotations which were considered exemplar for the themes were chosen and discussed within the research team. Descriptive statistics were performed to describe the sociodemographic characteristics of participants. Characteristics were compared between relatives and individuals from the general population using independent sample T-tests for continuous data and Chi-square tests for categorical data. The Statistical Package for Social Sciences (SPSS) version 25.0 was used for all statistical analyses (SPSS, Inc., Chicago, IL, USA).

## Results

### Participants

Sociodemographic characteristics of participants and their familiarity with carrier testing are shown in Table 1. In total, 23 relatives of MPS III patients participated including eighteen parents (78.3%), two siblings (8.7%), two aunts (8.7%), and one grandfather (1%). Individuals from the general population (N=18) were significantly younger and more often considered a (future) pregnancy. Relatives of MPS III patients were more often aware of the Amsterdam ECS for 50 severe genetic disorders compared to participants from the general population, due to the fact that most relatives had also participated in a previous questionnaire study on ECS (Nijmeijer et al. 2020).

**Table 1:** Characteristics of participants in the focus groups

	All participants N=41 (%)	Relatives N=23 (%)	General population N=18 (%)	p*
Age in years; mean (SD)	43.0 (15.63)	53.9 (12.73)	29.7 (4.45)	**<.001**
Age categories				
18−24	2 (4.9)	0 (0.0)	2 (11.1)	
25−34	14 (34.1)	1 (4.3)	13 (72.2)	
35−45	9 (22.0)	6 (26.1)	3 (16.7)	
46−76	15 (36.6)	15 (65.2)	0 (0.0)	
Female gender	22 (53.7)	12 (52.2)	10 (55.6)	.83
Country of birth (Netherlands)	40 (97.6)	22 (95.7)	18 (100.0)	.37
Educational level^a^				.71
Low	4 (9.8)	3 (13.0)	1 (5.6)	
Intermediate	16 (39.0)	9 (39.1)	7 (38.9)	
High	21 (51.2)	11 (47.8)	10 (55.6)	
Religious beliefsb				.13
No	20 (48.8)	10 (43.5)	10 (55.6)	
Yes	19 (46.3)	13 (56.5)	6 (33.3)	
I do not want to say	2 (4.9)	0 (0.0)	2 (11.1)	
Marital status				**.01**
Single	7 (17.1)	1 (4.3)	6 (33.3)	
In a relationship/married	34 (82.9)	22 (95.7)	12 (66.7)	
Have child(ren)				**.001**
No	12 (29.3)	2 (8.7)	10 (55.6)	
Yes	29 (70.3)	21 (91.3)	8 (44.4)	
Considering a (future) pregnancyc				**<.001**
No	19 (46.3)	19 (82.6)	0 (0.0)	
Yes	22 (53.7)	4 (17.4)	18 (100)	
Have you ever heard of the carrier test for 50 genetic disorders before this focus group?				**<.001**
No	19 (46.3)	3 (13.0)	16 (88.9)	
Yes	22 (53.7)	20 (87.0)	2 (11.1)	
Have you ever taken a carrier test?				**<.001**
No	32 (78.0)	14 (60.9)	18 (100)	
Yes	9 (22.0)	9 (39.1)	0 (0.0)	

*Sociodemographic characteristics were compared between relatives of MPS III patients and individuals from the general population. Significant differences (*p*<.05) are presented in bold

^a^Educational level. Low: primary education, lower vocational education, lower and middle general secondary education. Intermediate: middle vocational education, higher secondary education, pre-university education. High: higher vocational education, university

^b^“Yes” if answers comprised the following: “active religious,” “a little active religious,” “religious, but not active”

^c^“Yes” if answers comprised the following: “I have no children at the moment but I would like to have children,” “I have children and my partner and I would like to have more children,” “I am/my partner is currently pregnant,” or “I would have liked to have children but I remained childless”

### Focus group results

Five evident themes were generated from the data: (1) benefits of ECS, (2) barriers to opt for ECS, (3) disclosure of test results: offering a choice, (4) severity as key criterion for ECS panels, and (5) support for ECS. Results for each theme are described below.

### Benefits of ECS

During the focus group discussions, participants reported various benefits of ECS. All arguments in favor of ECS were grouped in four overall categories relating to the interest for prospective parents, for the future child, for the family, and for society (illustrative quotes in Table 2). The potential benefit of ECS mentioned by the majority of both relatives and individuals from the general population was that the wide range of reproductive options after ECS could increase prospective parents’ autonomy and could provide more assurance of having a healthy child.

**Table 2:** Benefits of ECS mentioned by relatives and the general population with representative quotes

Themes	Exemplar quotes
Interest for prospective parents	
Offers (reproductive) choices for future parents	If I have to tell my children why they should get tested, I would say: because you are able to choose if you want to intervene if there is something wrong with the foetus (parent, Gr#2)
Prevents suffering for the parents^a^	Nobody wants to survive their own child. We [parents of a child with MPS III] all woke up a thousand times at night hoping that it was just a dream [their child being severely ill], but unfortunately the nightmare was not over (parent, Gr#2)
Mentally or practically prepare for a child with a genetic disorder	But otherwise you are prepared for your child to be ill, maybe that will make a difference mentally (parent, Gr#3) You may have to work fewer hours because you should be able to take care of your child (general population, Gr#5)
Fear or regret^b^	If there is something wrong [with your child], and you realize you had the opportunity to reduce the risk (general population, Gr#6)
Information about your own health status^b^	Getting insight into the status of your own health [...], the test provides some sort of insight (general population, Gr#7)
Being prepared as partners^b^	Being on the same page in advance [...]That may prevent future arguing, because you both knew it beforehand (general population, Gr#5)
Interest for the future child	
Prevents suffering for the child (by preventing the birth of an affected child)	I think preventing suffering for such a child is the most important reason of all (general population, Gr#5)
Information that allows earlier diagnosis and/or treatment after birth^a^	If we had known the diagnosis from birth, we had acted very differently [towards the child]. So, then the test is also useful, if you choose not to intervene (parent, Gr#1)
Interest for the family	
Prevents suffering for the family (by preventing the birth of an affected child)^a^	I do not think it only prevents suffering for the child, but also for the whole family (parent, Gr#2)
Cascade testing	The test results offers you the opportunity to warn other family members; they may also be at increased risk (parent, Gr#3)
Interest for the society	
Saves costs for society	You can have a discussion about how socially responsible it is to bring a disabled child into the world when you consider the costs (relative, Gr#4)
To decrease the prevalence of genetic disorders	To prevent that every year in the Netherlands there are still some children born with the disorder (parent, Gr#2) That such diseases can be eradicated (general population, Gr#5)
Decreases the burden on the health care system^a^	If you prevent the birth of children with severe disorders, you will also relieve the burden of the healthcare system dramatically. Because, uh, we spend a lot of time in the hospital (parent, Gr#1)

Arguments are both mentioned by relatives of MPS III patients and individuals from the general population

^a^Argument only mentioned by relatives of MPS III patients

^b^Argument only mentioned by individuals from the general population. *Gr*, group

The [ECS] test allows you to make a choice in advance [...], you can choose alternative ways to get pregnant (general population, Gr#6)

Related to the interest of the future child, both groups stated that ECS may prevent suffering for the child.

If it was up to us, she [child with MPS III] was never born. She has to go through so much suffering (parent, Gr#3)

Only relatives of MPS III patients mentioned that an earlier postnatal diagnosis can be a benefit of ECS, and that ECS offers potential benefits for other family members as well, allowing to prevent suffering for, e.g., siblings of an affected child and offering the option for cascade testing. Although arguments related to potential interest for society were less frequently mentioned, and sometimes accompanied by emotions, saving costs for society by preventing the birth of a child with profound disabilities as well as the feasibility to decrease the prevalence of these disorders was mentioned in both groups.

It sounds very harsh, but those costs [for an affected child] indirectly pass on to the rest of the Netherlands, in what they have to pay for healthcare (general population, Gr#5)

### Barriers to opt for ECS

Barriers for the intended uptake of ECS mentioned by participants were allocated into six overall categories relating to a lack of awareness, lack of genetic knowledge, a lack of personal relevance, psychosocial impact, practical barriers, and ideology and beliefs (illustrative quotes in Table 3). Individuals from the general population indicated that they had never heard of the availability of ECS and consequently did not consider to participate yet. Moreover, there was little knowledge on the concept of inheritance and confusion about the difference with the non-invasive prenatal test (NIPT) was expressed.

**Table 3:** Barriers of ECS mentioned by relatives and the general population with representative quotes

Themes	Exemplar quotes
Lack of awareness	
Lack of awareness about the availability of ECS	Do people even know it [ECS test] exist? If it is not offered, or you have not heard about it, you automatically will not participate (parent, Gr#1) I think most people have not heard about the test. Why? What is the reason that it is not known yet? (general population, Gr#6)
Ignorance about health risks for offspring	I think you do not even realize that eh, when I was pregnant of my first child [healthy sibling] I never thought there could be anything wrong (parent, Gr#3) When I was pregnant of my first child, I was not concerned at all. Only fun and excitement [..] That is the unawareness that a lot of people have; babies are born healthy right? (general population, Gr#6)
Lack of genetic knowledge	What is the reason they do not include Down Syndrome in the [ECS] test? Why is that not possible? (general population, Gr#6)
Lack of personal relevance	
Absence of a genetic disorder in the family	Honestly if it [genetic condition] does not occur in your family or circle of friends, you will not think about it” (relative, Gr#4) There is no history of diseases known in my family (general population, Gr#7)
Low perceived risk	For me the risk feels negligible [for the other 49 disorders], it is 1:600 and I think that is approximately 0.17 percent? Well, 0.17 percent is negligible (relative, Gr#4) If you see so many healthy people and so few disabled people, it feels like you have a very small chance. [...] It just feels like 1:6000 instead of 1:600. I also think 1:600 is a pretty small chance, but if I compare it with something else like, uh, like the chance that an airplane will crash, then I would think, I would not fly (general population, Gr#5)
Psychosocial impact	
Test offer/results leads to anxiety and worries	That you are suddenly completely mentally confused, [...], that you may be a carrier of 10 disorders (parent, Gr#2)
Being confronted with difficult decisions	The dilemma of what to choose in case of a positive test result (relative, Gr#4) It is of course stressful, choices have to be made (general population, Gr#6)
Disagreement or friction with partner	Partner does not want to opt for ECS (parent, Gr#3) One partner wants to take the risk, , the other partner does not [...]. Then there will be conflicts (general population, Gr#5)
Practical barriers	
Costs of the test	As much as I would like to know, I cannot afford it. [...] We would have to save money to cover those extra costs (general population, Gr#6)
The test does not provide a 100% guarantee of having a healthy child	This test includes 50 disorders, there are many other disorders for which you may be at an even greater risk (relative, Gr#4) Even though they told you that nothing is wrong, it is not 100% certain (general population, Gr#5)
Ideology and beliefs	
Religious beliefs (reproductive options are not an option)	I would not opt for the test, but eh, that is only because of our religion (parent, Gr#3) If God wants your child to be ill you should leave it that way, some people might think (general population, Gr#5)
Every child is welcome^b^	Children with an illness also have the right to be born (general population, Gr#5)
Fear of eugenics “perfect baby”^a^	The risk of preparing a perfect human being (parent, Gr#1)
Unnatural^a^	It is unnatural. Not everything can be planned (relative, Gr#4)

Arguments are both mentioned by relatives of MPS III patients and individuals from the general population

^a^Argument only mentioned by relatives of MPS III patients

^b^Argument only mentioned by individuals from the general population. *Gr*, group

Well yes I could do the NIPT-test. But eeeh, the carrier screening test, haven’t heard about this. Isn’t this test similar to NIPT? (general population, Gr#7)

Relatives mentioned that the absence of a genetic disorder in the family would probably be the most important barrier for prospective parents *without* experiential knowledge. Indeed, most participants from the general population evaluated ECS as less relevant for this reason. Some participants perceived the reported risk of having affected offspring (1:600) as low, which led to discussions with others who disagreed.

I am healthy, my partner is healthy. Nobody in the family has a disorder. So, I assume in my next pregnancy that the child will be healthy as well [...] all reasons why I should not opt for the test, I guess (general population, Gr#7)

Anxiety and worries about the test results were also mentioned. Some participants preferred a carefree pregnancy or were afraid that they would no longer dare to conceive, which would be a reason for them to refrain from ECS.

I believe that if you know [that you are a carrier] in advance, that it can drive you crazy if you think about it too much (general population, Gr#5)

The costs of the test (e.g., 650 euro for the Amsterdam UMC test) were mentioned as a practical barrier and were extensively debated. Both groups expressed the concern that these costs may lead to inequality in access since many people can probably not afford this and they held the opinion that health insurances should (partly) reimburse these costs. Relatives, however, reasoned from an experiential perspective that the costs for ECS compare highly favorable to the extra costs involved in caring for a child with a severe genetic disorder. Regarding the category “ideology and beliefs,” a concern expressed by a relative was that genetic technologies might be used to create a world with only perfect people (eugenics).

Then it is a problem that people who can afford it will opt for the test, and people who do not have that amount of money will not (relative, Gr#4)

### Disclosure of test results: offering a choice

Participants discussed the pros and cons of both full disclosure of individual test results or only disclosing couple-based test results. Most arguments were similar for relatives and the general population (illustrative quotes in Table 4). Most participants preferred full disclosure of individual test results. In particular, having access to information that can be of importance to the future child or other family members (cascade testing) was considered an important advantage.

**Table 4:** Mentioned arguments regarding full disclosure of test results versus couple-based test results

Themes	Exemplar quotes
Full disclosure of test results	
Important information for children and other family members (cascade testing)	I would like to know if I am a carrier, because my child could also be a carrier (general population, Gr#5)
Avoid testing again in case of a divorce/split up with partner	I would prefer receiving my own result, because things can go wrong [in case of relationships] and then you would have to test yourself again if you have another partner (relative, Gr#4)
It is my right to get all results	I mean, you both give your blood as individual [for the test], so I think that you also should get your results individually. [...], but if you pay E650,- out of pocket you want to know everything (general population, Gr#7)
Curiosity	I am just curious about the result. You do not choose to do this test for no reason. When I would do this test, I would like to know if I am a carrier (parent, Gr#2)
Relevant for sperm donor or woman who want to freeze their eggs	Women [with a desire to conceive] who do not have a relationship yet, and for example want to freeze their eggs or need a sperm donor, would also want to know if they are a carrier. And maybe it is also relevant to test the donor then (general population, Gr#6)
Couple-based test results	
Individual information leads to anxiety	If you are very anxious, if you are sensitive for that [being anxious], you might worry about suffering [from one of the diseases] (general population, Gr#5)
Consequences of being both carrier of the same disease is only relevant	I would opt for this [couple-based] because what is the purpose of knowing all those diseases? You are only interested in the match (parent, Gr#3)
Commitment to partner	I would choose as a couple, because, well, there is your risk, in that match. Call me a romantic, but you sit there as a couple, as a couple you want a child […] And it is better not to know what you do not need to know (relative, Gr#4)

I would like to tell that information to my children, because, I think it is important that they get tested too (parent, Gr#3)

Furthermore, participants emphasized the importance of individual test results when changing partners as it was considered undesirable to have and pay for another test in case of a new partner. Moreover, “the right to know” as much as possible about your own biological material or when having paid for the test out-of-pocket, was suggested as reasons in favor of reporting individual test results.

Some participants expressed a preference not to be burdened by individual test results because it might cause unnecessary anxiety about one’s own health. Another reason to prefer couple-based test results was feeling a commitment towards the partner because “as a couple” you embark on the adventure of having children.

Participants in several groups suggested to give people the choice between individual results or couple-based test results to increase their autonomy. Additionally, it was suggested to offer full disclosure of test results at higher costs.

Maybe it is a solution to give people the choice themselves which option they prefer. Because if one partner does not want to know all the information, but the other does, then you can choose (general population, Gr#1)

### Severity as a key criterion for ECS panels

All participants agreed that childhood-onset and life-limiting disorders for which no disease-modifying treatment is available should be included in ECS panels. Disease severity was often mentioned as the criterion to decide which types of disorders (e.g., only physical disabilities or intellectual disabilities or both) should be included in screening panels. Relatives mentioned that it can be challenging to determine if disorders are “severe enough” to include in screening panels as some genetic disorders, including MPS III, are associated with a variability of disease expression and you cannot always reliably predict the course of the disease before birth.

The first time we met a couple who’s child also had MPS III, the mother came up to us immediately and told us that her child still took swimming lessons and everything, while our son couldn’t do this at all. Our son is now 25, while her child has become no older than 16. And then I think, how is that possible? [...] It cannot be predicted (parent, Gr#1)

The perceived severity of a disease, according to participants, was related to (1) self-sustainability; (2) if the child would be in pain; (3) if the child would have to undergo medical procedures; and (4) if “the child could really be a child.” It was noticeable that individuals from the general population more often classified disorders as severe and suitable to include in ECS panels compared to relatives. For example, relatives classified other disorders, such as congenital blindness and deafness, as relatively mild compared to the severity of MPS III and considered such disorders not severe enough to be included in the panel, while participants from the general population believed they could be included.

All participants expressed serious doubts concerning the inclusion of late-onset disorders in screening panels. Some participants said that late-onset disorders are part of life and that one should not try to play for God. Others stated that they did not want to deprive their child of a carefree childhood.

On the one hand, some participants supported the possibility that one should be free to select which disorders or categories of disorders one would like to have included in an ECS panel.

In my opinion, you can offer it [optional disorders and categories of disorders] and then everyone can decide for themselves (general population, Gr#1)

On the other hand, other participants were concerned that free choice of disease (categories) might lead to unwanted “designer babies.”

No, then you go shopping [for which disorder you prefer screening]. I find this very difficult to consider. It shouldn’t be a lottery. Then you go back to that designer [baby] argument. Well, I think that, I think that’s too much (parent, Gr#1)

### Support for ECS

All participants had a positive attitude about ECS and considered the offer of such a test important for all prospective parents. Moreover, they believed that ECS should be made available more actively, for example by health professionals such as the general practitioner and via specialists. To increase awareness about ECS, participants believed the government should have a leading role and suggested to make use of commercials, banners, social media, information on contraceptives, and flyers. Moreover, high school was mentioned as setting in which teenagers could be informed for the first time about the availability of ECS.

Maybe first a big national campaign and after this, smaller initiatives should be initiated to keep the information up-to-date. I think the majority of people does not know anything [about ECS] (general population, Gr#5)

## Discussion

This study explored the perspectives of relatives of MPS III patients and members of the Dutch general population on several important aspects related to preconception ECS. As the current study used a qualitative focus group design, we were able to further assess the motives, considerations, and feelings of relatives of MPS III patients and the general population about ECS, related to the opinions as reported in earlier survey studies on ECS (Nijmeijer et al. 2019; Nijmeijer et al. 2020).

Despite initial confusion about its purpose, both relatives of MPS III patients and individuals from the general population supported an offer of ECS to all prospective parents in the Netherlands. Participants mentioned the benefits of ECS for prospective parents, but also emphasized the benefits from other perspectives (i.e., the future child, family, and society). These positive views are in agreement with the results of earlier survey studies on the attitudes of relatives of MPS III patients (Nijmeijer et al. 2020) and the general population (Nijmeijer et al. 2019; Ong et al. 2018; Plantinga et al. 2016; Van Steijvoort et al. 2020). Participants in our study believed that the population needs to be more actively informed about the availability of ECS, which underpins their positive attitudes.

Discussing the benefits of ECS, only relatives of MPS III patients mentioned an earlier postnatal diagnosis as a possible benefit of ECS. This outcome is likely related to a long diagnostic delay experienced by many parents of MPS III children (Kuiper et al. 2018). Furthermore, only relatives mentioned benefits of ECS for other family members, as they know from experiential perspective that a severe genetic disorder may have significant psychosocial impact on the whole family (Somanadhan and Larkin 2016).

Despite positive attitudes, participants mentioned significant barriers for implementation. The out-of-pocket costs associated with ECS testing were seen as an important barrier to participate in ECS and could result in inequality in access. Currently, ECS for prospective parents in the Netherlands without a medical indication, for example a positive family history, belonging to an ancestry-based or geographically based high-risk group, is not reimbursed by health insurance companies. Previous studies in the Netherlands (Nijmeijer et al. 2019; Plantinga et al. 2016), Australia (Ong et al. 2018), and the UK (Briggs et al. 2017) showed that most prospective parents are willing to pay a maximum of approximately 50–150 euro, whereas the current costs of ECS in many countries worldwide are much higher. In accordance with previous studies (Gilmore et al. 2017; McClaren et al. 2008; Nijmeijer et al. 2019; Nijmeijer et al. 2020), a lack of perceived personal relevance due to the absence of a genetic disorder in the family was believed to be an important reason for people to refrain from having ECS. Initiatives to inform couples planning a (near) future pregnancy about the rationale of ECS (e.g., aiming at couples without positive family history), for example with public education or awareness campaigns, are essential and were encouraged by all participants in our study. In line with our findings, Holtkamp et al. (2017) showed that Dutch key stakeholders including those working in the field of carrier screening believed that more awareness of ECS and genetic disorders included in screening panels could overcome a lack of demand among the general public as well.

Concerns about social stigmatization or discrimination are frequently mentioned in discussions about the acceptability of carrier screening (van der Hout et al. 2016). Although offering ECS to the general population might reduce stigmatization of specific ethnic groups compared to ancestry-based panels, expanded screening panels could reinforce the stigmatization of disabilities in general (Parens and Asch 2003; van der Hout et al. 2016). In the current study, however, relatives of MPS III patients did not mention concerns about any form of stigmatization. The strong positive attitudes of MPS III relatives towards ECS may be related to the neurodegenerative and progressive course of MPS III, which places a significant burden on the child and the family (Conijn et al. 2018; Grant et al. 2013). Boardman et al. (2017) showed in their study on the views of affected families towards population screening for SMA that attitudes towards screening were highly correlated with quality of life (QoL) rated by patients themselves or by their family members. Those perceiving the lowest QoL were most likely to support the implementation of ECS for SMA compared to those perceiving a good QoL.

In all focus groups, the majority of participants preferred full disclosure of individual test results instead of couple-based results. This is in accordance with previous studies on the attitudes towards ECS (Nijmeijer et al. 2019) and carrier screening for CF (Henneman and Ten Kate 2002). An innovative suggestion of participants in the current study was to give (prospective) parents the choice. However, such a choice option may be challenging in practice. Moreover, full disclosure of test results may be less feasible when screening for a high number of variants due to the complexity and costs of providing (post-test) counseling for a relatively high amount of disorders, as it is estimated that every individual is a carrier of ~ 20 AR disorders (Antonarakis 2019). Couple-based test results are expected to reduce the impact on workload and costs in the laboratories, and the amount of work and costs associated with subsequent genetic counseling (Lynch et al. 2018; Schuurmans et al. 2019). However, the limited possibility for cascade testing in families in case of couple-based test results can also be perceived as a disadvantage, in particular for more common disorders such as CF (Kirk et al. 2019). This drawback may be overcome by additionally communicating only those disorders with higher carrier frequencies on an individual basis, as recently implemented in Belgium (UZ Leuven 2020). All participants agreed that severe childhood-onset disorders which substantially impair QoL, such as MPS III, should be included in ECS panels. In accordance with recommendations of the European Society of Human Genetics (Henneman et al. 2016) and a recent study on the development of the gene panel for the Australian Reproductive Genetic Carrier Screening Project (Kirk et al. 2020), disease severity was the most important characteristic when considering disorders suitable for screening.

Individuals from the general population more often classified disorders as severe and suitable to include in ECS panels compared to parents of MPS III patients. A discrepancy in perceptions of disease severity was also found in the study of Boardman et al. (2018), in which the general public had a more negative view of SMA compared to families themselves. The influence of experiential knowledge on perceptions of disease severity should be considered when drafting future policy decisions regarding the composition of ECS panels.

Opinions in the focus groups were divided on whether to give prospective parents the opportunity to choose from a list of individual disorders or to choose from different panels, for example categories of disorders similar in type or severity as suggested by Kraft et al. (2018). Genetic professionals in the study of Chokoshvili et al. (2016) believed that offering individual couples a choice of disorders for which they would like to be screened is not feasible in a population screening setting as it may be complicated for prospective parents without prior experience to understand individual disorders. This is in accordance with a previous study assessing health professionals’ preferences towards the NIPT offer (Tamminga et al. 2015). Moreover, many genetic disorders included in ECS panels have a wide variation in severity which cannot be predetermined with ECS. Counselling potential users on all individual disorders and their phenotypic variation would not be feasible within the time currently available. As this might jeopardize informed decision-making, a model of generic consent has been proposed to meet this challenge (Dondorp et al. 2012.

Some limitations of the current study need to be discussed. First, the perspectives of relatives of MPS III patients may not be generalizable to relatives of patients with other genetic disorders included in ECS panels, as previous studies suggest that disease severity affects the attitudes of patients and their family members towards preconception ECS (Boardman et al. 2019; Boardman et al. 2017). Second, more than half of the participants were highly educated which is not a reflection of the general population in the Netherlands. However, the association between educational level and interest in ECS is unclear (Chokoshvili et al. 2017; Gilmore et al. 2017; Nijmeijer et al. 2019; Ong et al. 2018; Plantinga et al. 2016). Third, response bias may have occurred if relatives who support ECS implementation and who previously participated in the survey study were more likely to attend the focus groups. Although many relatives of MPS III patients already participated in the previous survey study on ECS, we did not observe any differences in their understanding or attitudes compared to relatives who did not. Fourth, the way in which we explained the concept of ECS to members of the general population and relatives of MPS III patients was not equal as relatives of MPS III patients were not shown the educational video (Conijn et al. 2020). Since relatives were shown a short presentation on the concepts of inheritance included in the educational video and presumably had more genetic knowledge at baseline than the general population (Nijmeijer et al. 2020), bias likely has been kept to a minimum. Finally, participants with stronger voices might have dominated the discussion resulting in “false consensus,” which we aimed to avoid by carefully moderating the discussions.

## Conclusion

In conclusion, this focus group study provided valuable insights that can enrich the discussions on the implementation of preconception ECS. It is shown that both relatives of MPS III patients and individuals from the Dutch general population support further implementation of preconception ECS to prospective parents without an a priori increased risk, especially for severe, childhood-onset disorders. However, important barriers for ECS implementation were identified. The concept of ECS was difficult to grasp for participants, especially for individuals from the general population. Educating prospective parents about the concept of ECS and the type of disorders included in ECS panels is essential for creating awareness and ensuring an informed choice when offering ECS.

## Data Availability

The datasets generated during and/or analyzed during the current study are available from the corresponding author on reasonable request.

## Declarations

The authors confirm independence from the sponsors. The content of this article has not been influenced by the sponsors.

### Animal studies

This article does not contain any studies with animal subjects performed by any of the authors.
